# Increased Fibrosis and Interstitial Fluid Pressure in Two Different Types of Syngeneic Murine Carcinoma Grown in Integrin β3-Subunit Deficient Mice

**DOI:** 10.1371/journal.pone.0034082

**Published:** 2012-03-30

**Authors:** Tomas Friman, Renata Gustafsson, Linda B. Stuhr, Jean Chidiac, Nils-Erik Heldin, Rolf K. Reed, Åke Oldberg, Kristofer Rubin

**Affiliations:** 1 Department of Medical Biochemistry and Microbiology, Uppsala University, Uppsala, Sweden; 2 Department of Experimental Medical Science, Lund University, Lund, Sweden; 3 Department of Biomedicine, University of Bergen, Bergen, Norway; 4 Department of Immunology, Genetics and Pathology, Uppsala University, Uppsala, Sweden; Dresden University of Technology, Germany

## Abstract

Stroma properties affect carcinoma physiology and direct malignant cell development. Here we present data showing that α_V_β_3_ expressed by stromal cells is involved in the control of interstitial fluid pressure (IFP), extracellular volume (ECV) and collagen scaffold architecture in experimental murine carcinoma. IFP was elevated and ECV lowered in syngeneic CT26 colon and LM3 mammary carcinomas grown in integrin β_3_-deficient compared to wild-type BALB/c mice. Integrin β_3_-deficiency had no effect on carcinoma growth rate or on vascular morphology and function. Analyses by electron microscopy of carcinomas from integrin β_3_-deficient mice revealed a coarser and denser collagen network compared to carcinomas in wild-type littermates. Collagen fibers were built from heterogeneous and thicker collagen fibrils in carcinomas from integrin β_3_-deficient mice. The fibrotic extracellular matrix (ECM) did not correlate with increased macrophage infiltration in integrin β_3_-deficient mice bearing CT26 tumors, indicating that the fibrotic phenotype was not mediated by increased inflammation. In conclusion, we report that integrin β_3_-deficiency in tumor stroma led to an elevated IFP and lowered ECV that correlated with a more fibrotic ECM, underlining the role of the collagen network for carcinoma physiology.

## Introduction

In addition to the malignant cells a carcinoma contains a connective tissue compartment, or stroma, which constitutes the microenvironment for the malignant cells. Malignant cells must initiate or alternatively find a permissive microenvironment in order to establish themselves and grow. The microenvironment extracellular matrix (ECM) provides a scaffold for tumor growth and blood supply. The stroma typically exhibits distorted blood vessels, hypoxia, and acidic pH, as well as infiltrating myeloid cells and activated connective tissue cells that commonly produce a fibrotic ECM [1]–[4]. Formation of a stroma depends on signals from the malignant carcinoma cells and the non-malignant vascular, connective tissue and inflammatory cells. The stroma in turn influences the phenotype of the malignant cells [2]–[4]. Several experimental studies have pointed to the expression of collagen type I in the microenvironment and degree of cross-linking of collagen fibers as a determining factor in tumor progression including metastasis [4]–[6].

Interstitial fluid pressure (IFP) is one of the Starling forces [7] that control capillary-to-interstitium fluid transport. In normal loose interstitial connective tissue, the IFP is close to zero, or slightly negative, and will normally limit perturbations in fluid exchange. However, during anaphylaxis, inflammation and burn injuries, the properties of the loose connective tissue surrounding blood vessels is altered, reflected by a rapid reduction in IFP that induces, rather than prevents increases in extracellular volume (ECV) and subsequent edema formation [8]. Available data suggest that connective tissue cells apply tensile forces on ECM fibers, which, in turn, inhibit the under-hydrated ground substance from taking up fluid and swell. A decrease in cellular tension on the ECM fibers allows the ground substance to swell, *i.e.* to form edema. β_1_-integrins mediate the tensile forces from the cytoskeleton to the ECM in normal tissues. However, dermal IFP lowered after anaphylaxis can be normalized by instilments of PDGF-BB or insulin [9]–[11] by a mechanism dependent on integrin α_V_β_3_ instead of β_1_-integrins [12], [13]. Carcinomas are characterized by a pathologically high IFP, which leads to impaired uptake of anti-cancer drugs into the carcinoma [14]–[16]. Agents that lower IFP in experimental carcinoma, increase the uptake and efficacy of chemotherapeutic agents [17]–[20].

Inhibition of PDGF-B with an aptamer that specifically binds and immobilizes PDGF-B or, alternatively, inhibition of PDGF receptors with the selective tyrosine kinase inhibitor STI571 (Glivec/Imatinib), lowers IFP in experimental carcinoma. This has been shown to increase both capillary-to-interstitium transport of a low-molecular weight tracer and sensitivity to chemotherapeutic agents [21]. As α_V_β_3_ is activated downstream of stimulation by PDGF-BB or insulin during IFP regulation in dermis [12], [13], we reasoned that an investigation into whether integrin α_V_β_3_ is crucial for elevated IFP in tumors was warranted. Here, we have investigated properties of the stroma in carcinomas grown in mice lacking the integrin β_3_-subunit to test if the absence of integrin α_V_β_3_ signaling results in lowered IFP.

## Results

### The lack of integrin β_3_-subunit in stroma elevates interstitial fluid pressure in experimental carcinoma

**Figure 1:**
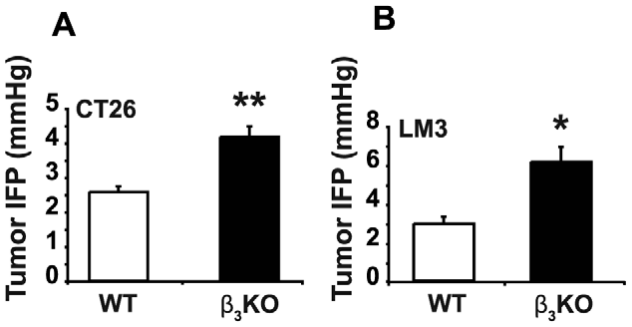
Interstitial fluid pressure (IFP) in CT26 and LM3 carcinomas. **A**) CT26 carcinomas. Data are from combined experimental series (n = 24 for WT; n = 21 for integrin β_3_-deficient mice). ** indicates p<0.001 by Student's t-test. Error bars are SEM. **B**) LM3 carcinomas. Data are from one experiment (n = 6 per genotype). * indicate p<0.05 by Mann-Whitney test. Error bars are SEM.

CT26 colon carcinoma and LM3 breast carcinoma cells formed tumors in integrin β_3_-deficient and WT mice with a 100% tumor take in both genotypes. LM3 breast carcinomas were grown in female mice, whereas CT26 carcinomas were grown at equal ratios in male and female mice. Tumor end-weights showed no significant difference in integrin β_3_-deficient compared to WT mice in either CT26 tumors or LM3 tumors (Figure S1A & B). The IFP in both CT26 and LM3 carcinomas was, however, significantly higher in β_3_-deficient carcinomas (Figure 1A and 1B). CT26 carcinomas had an average IFP of 4.2±0.3 mmHg when grown in integrin β_3_-deficient mice (n = 21) and 2.6±0.2 mmHg (p<0.001) when grown in WT mice (n = 24). The corresponding values for LM3 carcinomas were 6.1±0.8 and 2.9±0.4 mmHg (n = 6 for each genotype; p<0.05), respectively.

### The lack of integrin β_3_-subunit in stroma alters collagen architecture in experimental carcinoma

**Figure 2:**
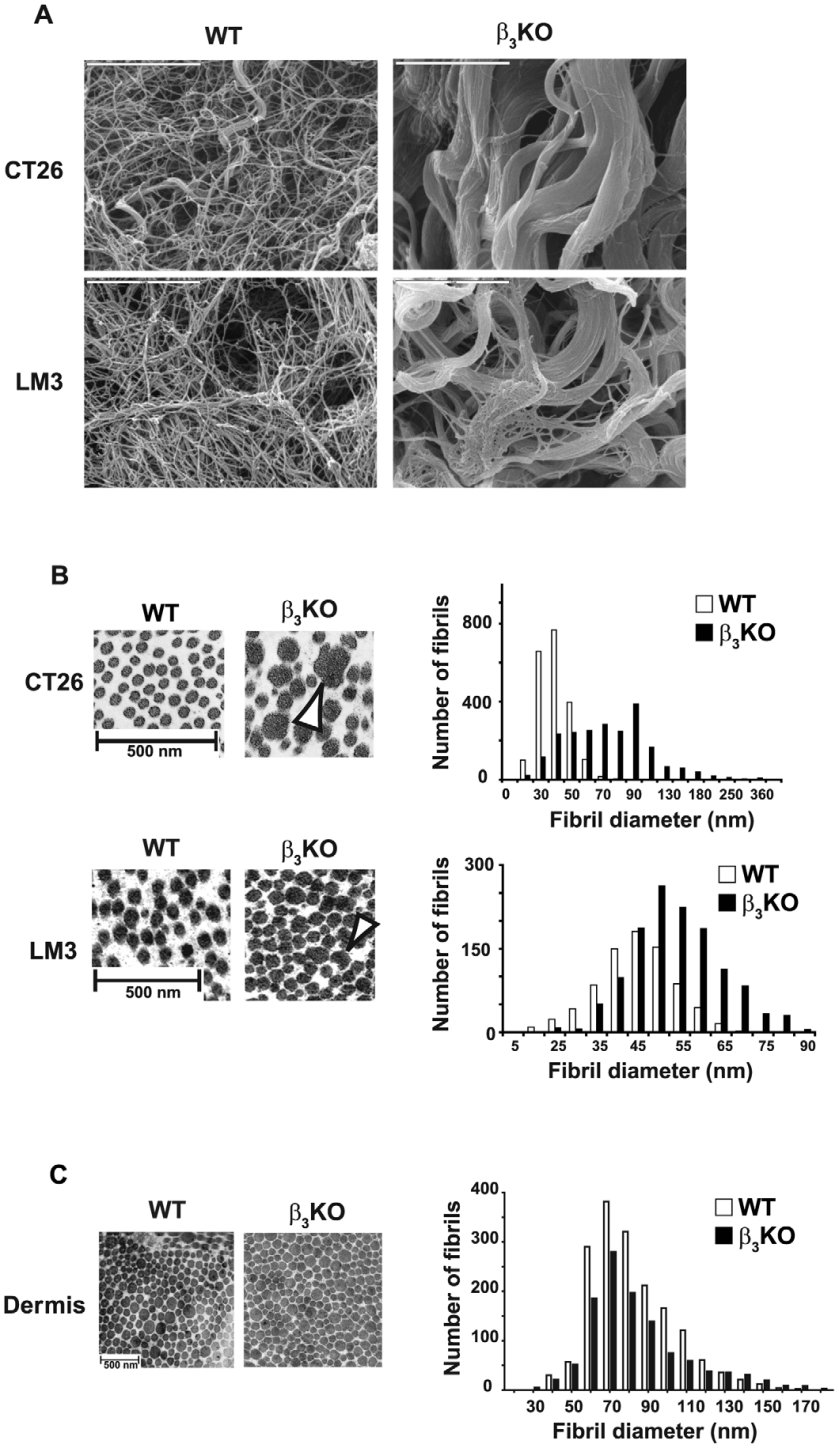
Analyses of collagen scaffold structure in CT26 and LM3 carcinomas grown in WT and integrin β_3_-deficient mice. **A**) Representative scanning electron micrographs at ×10,000 magnification revealed thicker, fused collagen fibers in both CT26 tumors (n = 5 per genotype) and LM3 tumors (n = 3 per genotype) grown in integrin β_3_-deficient mice (bars = 5 µm). **B**) Ultrastructural analysis of CT26 tumors (n = 5 per genotype) and LM3 tumors (n = 3 per genotype). Representative transmission electron micrographs on collagen fibril morphology demonstrated a heterogeneous fibril diameter distribution in CT26 tumors and LM3 tumors (at ×15,500 and ×11,500 magnification, respectively) grown in integrin β_3_-deficient compared to WT mice. Some of the fibrils were laterally fused (white arrowheads) in tumors grown in β_3_-deficient mice (bars = 500 nm). Histogram on collagen fibril distribution in CT26 tumors grown in integrin β_3_-deficient (black bars; n = 2159) and WT mice (white bars; n = 2043) and in LM3 tumors from integrin β_3_-deficient (black bars; n = 1286) and WT mice (white bars; n = 791), showing a clear shift toward thicker fibrils in both tumor types grown in integrin β_3_-deficient mice. **C**) Representative micrographs on tail dermis collagen fibrils from WT and integrin β_3_-deficient mice showed similar fibril distribution between the two genotypes (n = 2; bar = 500 nm). No significant difference could be observed in collagen fibril distribution from tail dermis derived from β_3_-deficient (black bars; n = 1157) or WT mice (white bars; n = 1715). To analyze fibril distribution, each data point (diameter for each fibril) was tabulated in GraphPad using the Frequency distribution analysis tool.

Ultrastructural analyses of the collagen scaffold by scanning electron microscopy (EM) at high magnification (×10,000) revealed striking differences in collagen fiber structure between carcinomas grown in β_3_-deficient and WT mice. In β_3_-deficient mice, both CT26 and LM3 carcinomas displayed a mixture of thin and thicker abnormally fused collagen fiber bundles whereas these features were limited in the WT counterparts (Figure 2A).

Analyses of collagen fibril structure by transmission EM revealed a heterogeneous distribution with laterally fused, cauliflower shaped fibrils in carcinomas grown in integrin β_3_-deficient mice (Figure 2B, white arrowheads) with significantly larger size compared to tumors grown in WT mice. To study the fibril diameter distribution for the total number of fibrils counted on the micrographs from CT26 tumors grown in integrin β_3_-deficient mice (n = 2159) and WT mice (n = 2043), each value for the fibril diameter (data point) were plotted in a histogram (Figure 2B). The fibril distribution in CT26 tumors from integrin β_3_-deficient mice was 57 nm in the 25^th^ percentile and 99 nm in the 75^th^ percentile, while it was 37 nm in the 25^th^ percentile and 50 nm in the 75^th^ percentile for the fibrils in tumors grown in WT mice. Hence, the histogram confirmed the uneven distribution of fibril diameters revealing a clear shift to thicker fibrils. Further analyses showed a significant increase (p<0.03) in the average fibril diameter in CT26 carcinomas grown in integrin β_3_-deficient mice (79±13 nm) compared to WT mice (44±2 nm) (n = 5 for each genotype; Figure S2A). The same analyses on the micrographs from LM3 tumors grown in integrin β_3_-deficient mice (n = 1286) and WT mice (n = 791) for each fibril counted also showed a clear shift to thicker fibrils in LM3 tumors from integrin β_3_-deficient mice with a fibrils distribution of 49 nm in the 25^th^ percentile and 62 nm in the 75^th^ percentile, while it was 41 nm in the 25^th^ percentile and 53 nm in the 75^th^ percentile for the fibrils in tumors grown in WT mice (Figure 2B). In addition, a significant increase (p<0.05) was found in the average fibril diameter in LM3 carcinomas grown in integrin β_3_-deficient mice (56±2 nm) compared to WT mice (47±2 nm) (n = 3 for each genotype; Figure S2B). In contrast, ultrastructural analyses of tail tendons, dermis and Achilles tendons showed no differences in collagen fibrils between the genotypes (Figure 2C and data not shown). No significant difference could be observed in collagen fibril distribution from tail dermis derived from β_3_-deficient (n = 1157) or WT mice (n = 1715) (Figure 2C) or in average fibril diameter in β_3_-deficient (81±7 nm) and WT dermis (81±6 nm) (n = 2 for each genotype; p>0.98; Figure S2C).

**Figure 3:**
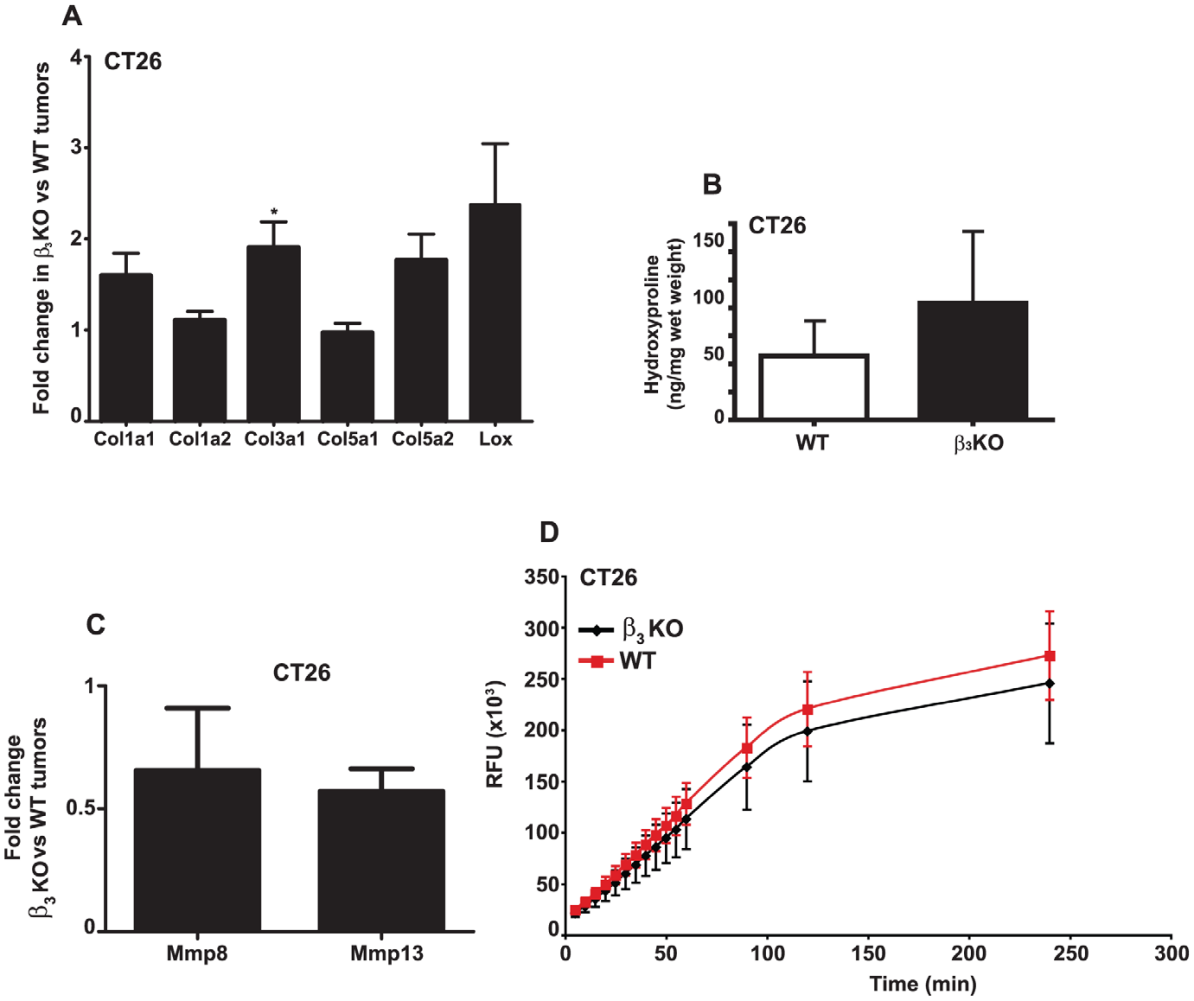
Quantitative RT-PCR analysis, hydroxyproline measurements and assay for MMP-13 activity. **A**) Relative mRNA expression of fibrillar pro-collagens and lysyl oxidase (Lox) in CT26 carcinomas from integrin β_3_-deficient (n = 12) compared to WT mice (n = 11). Bars indicate fold changes in tumors from β_3_-deficient mice after normalization to tumors from WT mice using the 2^−ΔΔCt^ method. * indicates p<0.05 with Student's t-test. Error bars are SEM. **B**) Hydroxyproline content in CT26 carcinomas grown in integrin β_3_-deficient and WT mice (n = 4 per genotype). Data was analyzed with Student's t-test. Error bars are SEM. Differences in collagen content were not significant. **C**) Fold change in relative mRNA expression for the collagenases Mmp8 and Mmp13 in CT26 carcinomas from integrin β_3_-deficient (n = 12) compared to WT mice (n = 11). **D**) CT26 tumor homogenates of each genotype were subjected to a MMP assay (n = 6). Recombinant MMP-13 and samples were activated by 4-aminophenylmercuric acetate for 40 min at 37°C. Fluorescence resulting from the cleavage of the substrate 5-FAM/QXL™520 FRET peptide was measured. Data was analyzed with Student's t-test. Error bars are SD.

Increased collagen fiber thickness and fibril diameter in tumors from integrin β_3_-deficient mice could be an indicator of elevated production of collagen. Indeed, there was a trend towards increased mRNA transcription levels of the fibrillar pro-collagen chains for Col1a1 and Col5a2, and a significant increase in Col3a1 in integrin β_3_-deficient CT26 tumors (p<0.05) when compared to CT26 tumors grown in WT mice (Figure 3A) as determined by quantitative real-time PCR (qRT-PCR). In addition, elevated expression of the collagen cross-linking enzyme lysyl oxidase (Lox) was detected in integrin β_3_-deficient CT26 tumors (Figure 3A). A similar increase in fibrillar pro-collagens and Lox mRNA levels were observed in LM3 tumors grown in integrin β_3_-deficient mice when compared to LM3 tumors grown in WT mice (Figure S3).

Analyses of collagen content in CT26 carcinomas by hydroxyproline quantification showed a trend towards a higher collagen content in carcinomas grown in integrin β_3_-deficient compared to WT mice (Figure 3B).

The increased production of collagen and the abnormal fusion of the collagen fibrils and fibers could also indicate an altered activity of collagenase enzymes. Although decreased mRNA levels of matrix metalloproteinases (Mmp) −8 and −13 were observed in CT26 carcinomas from integrin β_3_-deficient compared to WT mice (Figure 3C), there was no significant difference in tumor collagenase activity between the two genotypes (Figure 3D).

### The lack of integrin β_3_-subunit in stroma has no effect on vascular density, pericyte coverage or blood vessel function

**Figure 4:**
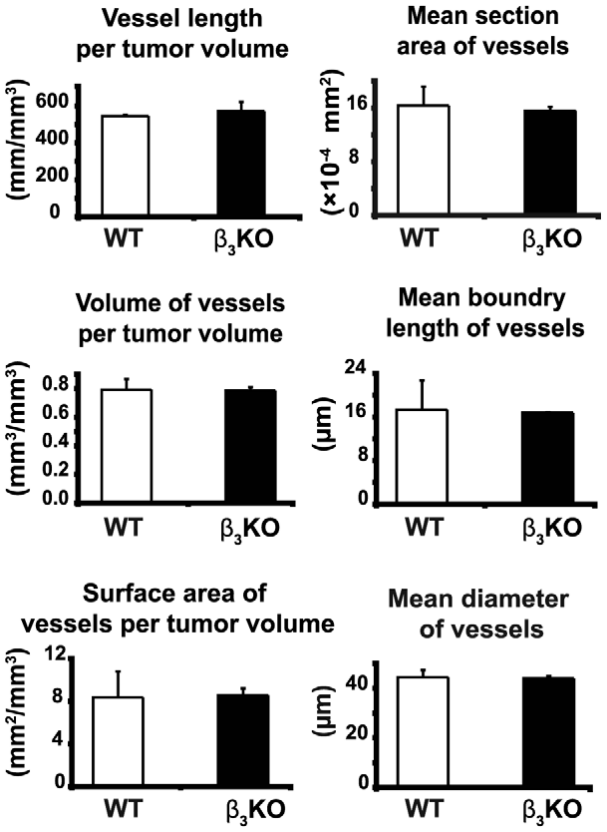
Quantitative analyses of vascular parameters. Stereology of CD31-positive vessels in CT26 carcinomas grown in integrin β_3_-deficient (n = 9) and WT mice (n = 6). 6 µm sections stained for CD31 by immunohistochemistry were counted, ×200 magnification. Data was analyzed with Student's t-test. Error bars are SEM.

**Figure 5:**
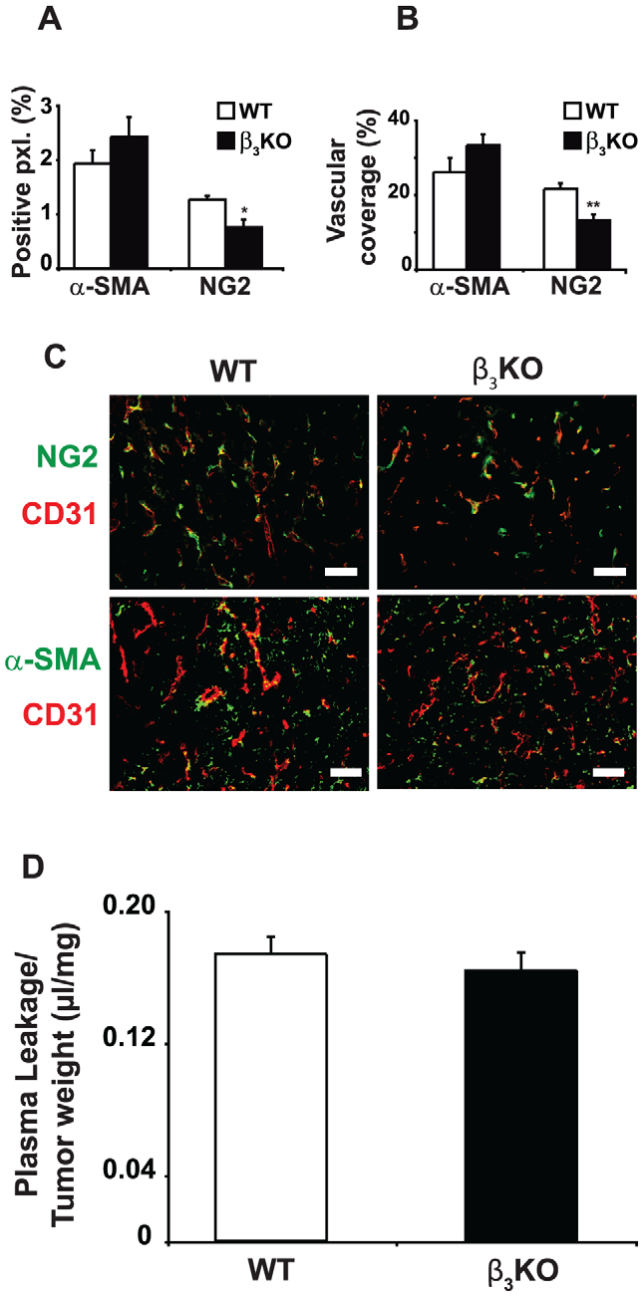
Pericyte coverage in CT26 carcinomas grown in integrin β_3_-deficient and WT mice. **A**) The total fraction of pxl. positive for NG2 or α-SMA were quantitated. **B**) The amount of CD31 and NG2 double positive pixels (pxl.) and CD31 and α-SMA double positive pixels were quantitated and presented as the fraction of CD31 pixels that were also NG2 or α-SMA positive. **C**) Representative immunofluorescence images of CD31 double stained with either NG2 or α-SMA in CT26 carcinoma sections from both genotypes (× 200 magnification). **D**) Leakage of Evans blue dye was assessed in integrin β_3_-deficient (n = 4) and WT mice (n = 5) bearing CT26 carcinomas. Leakage was corrected for the tumor plasma volume. Data are presented as leaked volume plasma per tumor tissue dry weight. Bar is 50 µm. * indicates p<0.05 and ** indicates p<0.01 by Mann-Whitney test. Error bars are SEM.

CD31 immunostaining showed an abundance of vessels in the viable zone of CT26 carcinomas grown in both integrin β_3_-deficient and WT mice but no significant differences in vessel density and morphology as assessed by stereology (Figure 4). Also, qRT-PCR analyses of mRNA expression from integrin β_3_-deficient and WT tumors showed equal levels of VEGF receptor-2 transcripts (Figure S4). Antibodies to the chondroitin sulfate proteoglycan NG2 were used to visualize activated pericytes. NG2 is known to be selectively expressed by pericytes in the vasculature of tumors [22]. Blood vessels in carcinomas grown in integrin β_3_-deficient mice displayed reduced NG2 positive staining and less co-localization between NG2 and CD31 (Figure 5A–C), but there were no significant difference in vascular coverage by α-SMA staining (Figure 5B and 5C). Hence, the pericyte coverage were similar between CT26 carcinomas grown in integrin β_3_-deficient and WT mice, however the phenotype of the pericytes differed between these two conditions. Additionally, α-SMA staining was not only confined to the vascular compartment, since also the extravascular compartment of the tumor stroma was positive for α-SMA staining (Figure 5C). There was no difference in total α-SMA staining in tumors from β_3_-deficient or WT mice (Figure 5A). Staining with reticular fibroblast marker showed no difference between the genotypes (data not shown), which supports equal content of carcinoma-associated fibroblasts. Vascular leakage of plasma proteins as determined by the modified Miles' assay, was similar in CT26 carcinomas grown in integrin β_3_-deficient and WT mice (Figure 5D). Moreover, the plasma volumes (PV) in CT26 carcinomas in the two genotypes were not significantly different, further supporting the hypothesis that vascular function was not affected by integrin β_3_-deficiency (Table 1). The extracellular volume (ECV) was, however, significantly lower in carcinomas grown in integrin β_3_-deficient mice (p<0.04), whereas total tissue water (TTW) was similar (Table 1). Collectively, the data show that the lack of integrin β_3_-subunit in tumor stroma has little effect on vascular function in CT26 carcinoma.

**Table 1:** Plasma volume (PV), extra cellular volume (ECV) and total tissue water (TTW) measurements in CT26 carcinomas and normal skin from integrin β_3_-deficient and WT mice.

	PV	ECV	TTW
	mL/g dry weight	mL/g dry weight	mL/g dry weight
Skin (WT) (n = 5)	0.029±0.007	0.778±0.158	1.836±0.995
Skin (β_3_KO) (n = 5)	0.039±0.018	0.643±0.123	1.657±0.331
Tumor (WT) (n = 8)	0.077±0.030	0.740±0.125	3.975±0.418
Tumor (β_3_KO) (n = 8)	0.094±0.051	0.472±0.252*	3.932±0.445

CT26 carcinomas (n = 8 per genotype) and skin (n = 5 per genotype).

* indicates p<0.04 between tumors from integrin β_3_-deficient and WT mice, determined with Student's t-test. Values are ± SEM.

### Reduced amounts of macrophages and inflammatory cytokines in tumors from integrin β_3_-deficient mice

**Figure 6:**
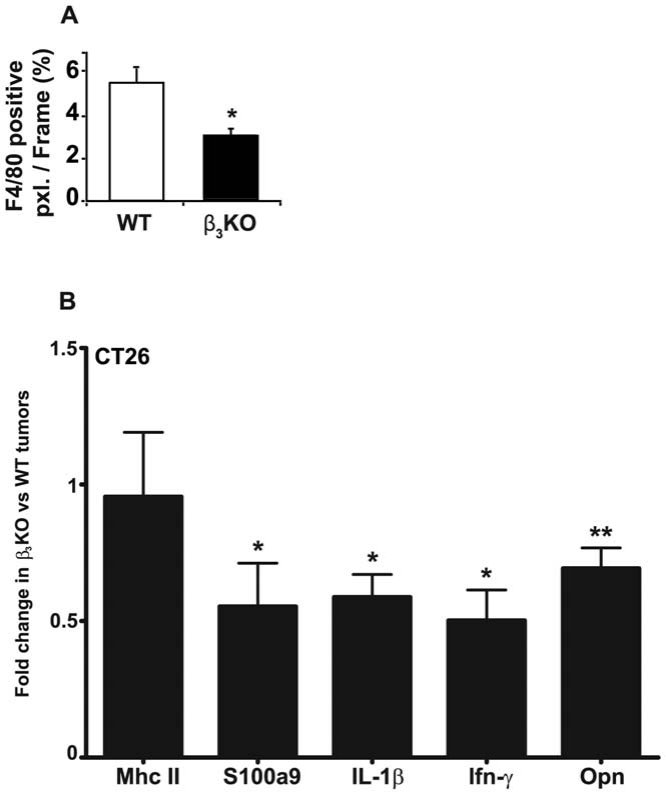
Inflammatory parameters in CT26 carcinomas. **A**) Macrophage content in CT26 carcinomas. The fraction of F4/80 positive pixels per image frame is presented (n = 7 for each genotype, ×200 magnification). * indicates p<0.05 by Mann-Whitney test. Error bars are SEM. **B**) QRT-PCR analysis of pro-inflammatory genes in CT26 carcinomas grown in integrin β_3_-deficient (n = 12) compared to WT mice (n = 11). Bars indicate fold changes in tumors grown in integrin β_3_-deficient mice after normalization to tumors from WT mice using the 2^−ΔΔCt^ method. * indicates p<0.04; ** indicates p<0.01 with Student's t-test. Error bars are SEM.

The numbers of F4/80 positive macrophages were significantly lower in CT26 carcinomas grown in integrin β_3_-deficient mice compared to tumors from WT animals (Figure 6A). However, there was no significant difference in the fraction of F4/80 staining that co-localized with MHC Class II staining (data not shown), which indicates that there was no difference in the fraction of macrophages with an activated phenotype. In close agreement with these results, qRT-PCR analysis indicated a down-regulation of pro-inflammatory transcripts in CT26 carcinomas grown in integrin β_3_-deficient compared to WT mice (Figure 6B), with significant decrease in S100A9, interleukin 1β (IL-1β), interferon-γ (Ifn-γ) and osteopontin (Opn) expression. However, there was no difference in MHC II mRNA expression.

## Discussion

We present data showing that absence of the integrin β_3_-subunit in the stroma of transplanted syngeneic LM3 mammary and CT26 colonic carcinomas results in a denser and coarser collagen network composed of thicker and irregular collagen fibrils. These qualitative changes in the collagen network architecture were paralleled by an elevated IFP. The present study thus support the hypothesis that IFP in carcinoma depend on qualitative properties of the stroma collagen network. We furthermore demonstrate that integrin β_3_-deficiency had no effect on the collagen scaffold architecture in dermis or tendons. The data advocate that β_3_-integrins have a role in determining collagen scaffold architecture in response to growth of malignant cells but are not involved in the buildup of normal connective tissue.

We have previously reported that PDGF-BB normalizes dermal IFP that has been reduced as a result of anaphylaxis and that this process critically depends on integrin α_V_β_3_ [23]. The control of IFP *in vivo* shares characteristics with collagen gel contraction *in vitro* and has been suggested to rely on modulation of tensile forces exerted by connective tissue cells on the ECM (reviewed in [8]). Furthermore, inhibition of PDGF-BB or PDGF-receptors lowers IFP in experimental carcinoma [18], [21]. This led us to hypothesize that carcinomas grown in integrin β_3_-deficient mice might have a reduced IFP compared to carcinomas grown in WT mice. However, the present data support a quite opposite conclusion, namely that in carcinoma, integrin α_V_β_3_ is not only dispensable for maintaining an elevated IFP but rather participates in processes that counteract increases in IFP. In this context it is worth noting that whereas PDGF BB-stimulated normalization of IFP in loose connective tissues occurs within minutes, reduction of carcinoma IFP by PDGF inhibitors requires days and is compatible with an effect on stroma architecture. A maintained high IFP in the tumor as observed here must mean that the hydraulic conductivity in the tumor interstitium is low providing a flow resistance to the drainage of fluid from the tumor interstitium towards the part of the tumor or tumor surrounding where functional lymphatics can remove the interstitial fluid.

Reduction of carcinoma IFP increases uptake and efficacy of low molecular weight cytostatic drugs in experimental carcinoma [17]–[19], [21], [24]. Inhibition of TGF-β1 and -β3 in xenograft carcinomas reduce IFP as well as the density of the collagen network in the tumors [25] and increases efficacy of cytostatic treatment [20]. Reduction of tumor fibrosis in a transgenic mouse model of pancreatic cancer has the same effect as lowering IFP on the uptake and efficacy of chemotherapeutics [26]. Furthermore, inhibition of collagen type I synthesis and deposition in experimental carcinoma, by treatment with losartan increase efficacy of nanotherapeutics [27]. Previous studies thus suggest that modulations of the collagen scaffold in carcinoma may be an effective way to increase efficacy of chemotherapy. Our present data opens for the possibility that functional down-regulation of integrin α_V_β_3_ could render carcinoma less accessible for chemotherapy by making the collagen scaffold less permissive for transport of solutes through the tumor tissue.

The relevance of the integrin α_V_β_3_ in tumor angiogenesis is uncertain [28], [29]. Mice lacking integrin β_3_ have been reported to support effective angiogenesis [30], [31] whereas pharmacological inhibition of α_V_β_3_ reduces angiogenesis in certain models [32]. The present data are consistent with reports showing that integrin α_V_β_3_ is not needed for tumor angiogenesis in that β_3_-deficiency had no significant effects on vessel density or morphology. Furthermore, integrin β_3_-deficiency had no significant effect on levels of mRNA encoding VEGF receptor-2. However, we recorded a difference in content and coverage of blood vessels by NG2-positive pericytes. On the other hand, α-SMA staining of vascular structures was similar between the two genotypes, indicating that there was no difference in pericyte coverage. The observed reduction in expression of NG2 by pericytes in β_3_ integrin-deficient tumors did not affect vascular density, plasma protein leakage or plasma volume. Together our results point to that the increased IFP in integrin β_3_-deficient tumors could not be attributed to any of the vascular parameters studied.

Differences in the vascular phenotype acquired by inhibition of VEGF receptor-2 or VEGF-A reduces IFP in experimental carcinoma [33], [34]. VEGF inhibition in KAT-4 carcinoma results in reductions of plasma protein leakage and ECV in parallel with a reduction of IFP [34]. In the present study, we observed that vascular leakage, a surrogate marker for vascular function, was not affected by the absence of integrin β_3_ in CT26 carcinomas, whereas ECV decreased and IFP increased. When taken together, available data counter the notion of a direct correlation between plasma protein leakage on the one hand, and ECV and IFP on the other. Treatment of experimental carcinoma with an inhibitor of PDGF receptors [35], with prostaglandin E_1_ [19] or growth of carcinoma in fibromodulin-deficient mice [25] generate tumors that have an increased ECV and reduced IFP. Our present data show that ECV was decreased and IFP increased in CT26 carcinomas grown in integrin β_3_-deficient animals, which fits with the aforementioned relation between ECV and IFP. If, however, plasma protein leakage is changed, as it appears to do after inhibition of the VEGF system [33], [34], this relation between ECV and IFP is not evident [34].

It has been reported that TGF-β signaling is enhanced during wound healing in integrin β_3_-deficient mice [36]. The increase in collagen deposition and network density reported here could have been due to an up-regulation of TGF-β signaling in CT26 carcinomas grown in β_3_-deficient mice. However, we did not detect any significant changes in mRNAs encoding TGF-β1, TGF-β3, TGFβR-II or connective tissue growth factor (CTGF) in CT26 carcinomas grown in integrin β_3_-deficient mice (Figure S4). CTGF expression reportedly is down-stream of TGF-β induced signaling [37] suggesting that the lack of stromal β_3_-integrin had little effect on TGF-β activation. Collectively, our data suggest that it is less likely that the effects on IFP and collagen architecture by integrin β_3_-deficiency are caused by an increased activity of TGF-β.

The reduction in macrophage content in CT26 carcinomas grown in integrin β_3_-deficient mice is consistent with earlier reports showing reduced macrophage infiltration in healing wounds [36] and experimental carcinoma [38] when integrin β_3_ is absent. The observed decrease in numbers of infiltrating macrophages may explain the decreased transcription levels of Mmp-8, Mmp-13 and Opn [39], [40]. The decreased expression of transcripts for the collagen degrading enzymes Mmp-8 and Mmp-13 may indicate perturbed collagenase activity, possibly affecting the architecture of the collagen scaffold in tumors. We were, however, unable to detect any difference in extractable MMP activity in lysates from β_3_-deficient and WT mice. Our data challenge the concept of a direct correlation between the degree of inflammation in carcinoma on the one hand and fibrosis on the other. It is possible that such a correlation exists in wild-type mice but that the absence of α_V_β_3_ decouples this relation. Further studies are needed to evaluate the relation between inflammation and fibrosis in carcinoma.

In conclusion, our data provide further support for a direct relationship between fibrosis and elevated IFP in carcinoma. Whether the increased fibrosis results from the disturbed fluid balance and hypertension in the carcinoma or if the fibrosis generates these disturbances in fluid maintenance in carcinoma is not clear.

## Materials and Methods

### Ethics Statement

All animal experiments were approved by the ethical committees for animal experiments in Uppsala, Sweden (permit number: C76/10) and in Bergen, Norway (permit number: 20102809).

### Tumor models

The BALB/c syngeneic tumor cell lines LM3 mammary carcinoma (kind gift of Dr. Elisa Bal de Kier Joffé, University of Buenos Aires, Argentina) [41] and CT26 mouse colon carcinoma (ATCC, Manassas, VA) were grown in RPMI 1640 (Invitrogen, San Diego, CA) supplemented with 10% Fetal Bovine Sera (Saveen-Werner, Limhamn, Sweden) and 60 µg/mL penicillin and 50 µg/mL streptomycin (SVA, Uppsala, Sweden). Fifty µL of cell suspension containing either 1.5×10^5^ CT26 cells or 5×10^5^ LM3 cells were injected into the left flank of BALB/c wild type or integrin β_3_-deficient mice [42]. The BALB/c integrin β_3_-deficient mouse strain was a gift from Richard O. Hynes, MIT, MA, USA. Integrin β_3_- deficient mice were bred heterozygous but prior to the experiments β_3_-deficient and WT mice lines were segregated. The mice were fed *ad libitum* and cared for regularly. CT26 tumors were grown for 3–4 weeks and LM3 tumors were grown 3–5 weeks. Upon sacrifice, all tumors were weighed before further analysis.

### Interstitial fluid pressure (IFP) measurements

Mice with tumors ranging in size between 0.5 and 1.5 mL were put on a heating pad under isofluran (Forene, Abbot Scandinavia, Solna, Sweden) anesthesia. The IFP was measured with the “wick-in the-needle” technique using 23 G needle as described [24]. At least three measurements were performed on every tumor. Four separate experiments were performed with CT26 tumors grown in integrin β_3_-deficient (n = 21) and WT mice (n = 24) and one with LM3 tumors grown in integrin β_3_-deficient and WT mice (n = 6 for each genotype). All mice were sex and aged matched in every experiment.

### Determination of plasma protein leakage in tumors

A modified Miles' assay was used, where Evans blue dye (EBD, 100 µL, 30 mg/kg body weight, Sigma, St Louis, MO) dissolved in 0.9% NaCl was injected *i.v.* into integrin β_3_-deficient (n = 4) and WT mice (n = 5) bearing CT26 tumors. After 30 min, blood was collected from the *inferior vena cava* and mice were subsequently sacrificed. The tumors were dissected and three pieces from each tumor was collected. The average piece weighed 371±158 mg. The tumor pieces were dried in a vacuum centrifuge. The dried tumor pieces were then incubated with 1 mL of formamide (Sigma) at 55°C for 16 hours. The amount of EBD in tumors and serum was quantified at 600 nm with a spectrophotometer (Titertek, Huntsville, AL). Measurements of EBD leakage were corrected for the tumor plasma volume (PV).

### Extracellular volume, plasma volume and total tissue water measurements

Extracellular volume (ECV) and plasma volume (PV) in CT26 tumors (n = 8 for each genotype) and skin (n = 5 for each genotype) were determined by the dilution principle using radioactive-labeled isotopes [34]. Total tissue water (TTW) was determined as (wet weight-dry weight)/dry weight. Tissue samples were dried at 50°C for 3–4 weeks until the weight was constant. The distribution volumes for ^51^Cr-EDTA (Institute of Energy Technology, Kjeller, Norway) and ^125^I-labeled human serum albumin (^125^I-HSA; Institute of Energy Technology), measuring ECV and PV respectively, were calculated as plasma equivalent volumes, *i.e.* counts per min (cpm) per mg of tissue divided by counts per min per mL of plasma. Both isotopes were given after functional nephrectomy by bilateral ligation of the renal pedicles via flank incision. ^51^Cr-EDTA (300,000 cpm in 0.2 mL PBS) was injected into the catheter in the tail vein and left to circulate for 85 min before injecting ^125^I-HSA (3×10^6^ cpm in 0.2 mL PBS). Blood samples were taken 5 min later by heart puncture. Skin from the back was used as a reference control. Radioactivity was determined in a COBRA II, Auto-gamma counter (Packard, Waltham, MA) with automatic background and spillover correction.

### Immunohistochemistry and immunofluorescence

Upon sacrifice, CT26 tumors were dissected and snap frozen in isopentane at −80°C. Frozen 6 µm sections were fixed in 4% paraformaldehyde (Merck, Darmstadt, Germany). Staining protocols were essentially as described [43]. Primary antibodies were as follows: monoclonal rat anti-mouse PECAM-1 clone Mec 13.3 and 390 (BD, San Diego, CA), polyclonal rabbit anti-mouse F4/80 (Serotec, Oxford, UK), monoclonal anti-mouse IA/I-E (clone 2G9) FITC conjugated (BD), polyclonal rabbit anti-mouse NG2 (Chemicon, Temecula, CA), mouse anti-α-smooth muscle actin (clone 1A4) FITC-conjugated (Sigma), rat anti-reticular fibroblast marker (Cederlane Laboratories, Ontario, Canada). The following secondary antibodies were used: goat anti-rat biotinylated or FITC conjugated and goat anti-rabbit Texas Red conjugated (Vector laboratories, Burlingame, CA). For immunohistochemistry, biotinylated immune-complexes were detected by the Vectastain ABC elite kit (Vector laboratories) with DAB as substrate (Zymed laboratories; Carlsbad, CA). Mayers Hematoxylin (Histolab, Göteborg, Sweden) and DAPI (Sigma) were used as a counter stains for immunohistochemistry and immunofluorescence respectively. Sections were analyzed with a Nikon Labophot fluorescence microscope and images were retrieved with a CCD camera and software from SPOT (Diagnostic Instruments, Sterling Heights, MI). Image analysis and quantifications was performed with Photoshop (Adobe, San José, CA) and ImageJ (NIH, Bethesda, MD) software. For quantification of pixels, at least 5 images (×200 magnification) per tumor (n = 7 for each genotype) for each type of staining were used. Quantification of blood vessels in CT26 tumors grown in integrin β_3_-deficient (n = 9) and WT mice (n = 6) was performed by stereology as described earlier [44].

### Hydroxyproline measurement

Hydroxyproline content was determined in CT26 tumor hydrolysates from integrin β_3_-deficient and WT mice (n = 4 for each genotype) essentially as described [45].

### RNA extraction and quantitative real time PCR (qRT-PCR)

Total RNA was extracted from CT26 tumors grown in integrin β_3_-deficient (n = 12) and WT mice (n = 11) and from LM3 tumors grown in integrin β_3_-deficient and WT mice (n = 6 for each genotype) with RNeasy Mini Kit (Qiagen, Valencia, CA) according to the manufacturers' instructions. RNA integrity was verified with electrophoresis and 1 µg total RNA was subjected to first strand cDNA synthesis with Superscript III First-Strand Synthesis Supermix for qRT-PCR (Invitrogen). QRT-PCR for each sample were performed on LightCycler (Roche Applied Science) in duplicates or triplicates by mixing cDNA with Maxima™ SYBR Green qPCR Master Mix (Fermentas, St. Leon-Rot, Germany) and gene specific primer pairs (available upon request). Amplification results were analyzed using LightCycler software Version 3. The calculated threshold cycle values for each gene were normalized to the threshold cycle value of the internal standard hypoxanthine phosphoribosyltransferase 1 (Hprt1). Relative gene expression levels are presented as fold change in tumors grown in integrin β_3_-deficient mice normalized to the gene expression levels in tumors from WT mice using the 2^−ΔΔCt^ method [46].

### Electron microscopy (EM)

Determinations of collagen fibril diameters were performed by transmission EM analyses of tail tendons, dermis and Achilles tendons from WT and integrin β_3_-deficient mice (n = 2 for each genotype); CT26 tumors (n = 5 for each genotype); and LM3 tumors (n = 3 for each genotype). Specimens were fixed in 0.15 M sodium cacodylate-buffered 2% glutaraldehyde, postfixed in 0.15 M sodium cacodylate-buffered 1% osmium tetraoxide, dehydrated in graded ethanol series, impregnated in acetone and embedded in epoxy resin. Ultra-thin sections from at least three different areas per sample were examined in a Philips CM-10 electron microscope (Philips, Amsterdam, Netherlands) and quantitated with ImageJ software (NIH). For scanning EM analysis to investigate fiber structure, CT26 tumors (n = 8 for each genotype) and LM3 tumors (n = 4 for each genotype) were processed by alkali maceration as described [47] and analyzed in a Philips 515 electron microscope.

### Matrix metalloproteinase assay

A Sensolyte® 520 matrix metalloproteinase (MMP) assay (Nordic Biosite, Stockholm, Sweden) was used according to the manufacturer's instructions. Briefly, CT26 tumors from WT and integrin β_3_-deficient mice (n = 6 for each genotype) were lysed overnight in the included assay buffer supplemented with protease inhibitors (Complete EDTA-free, Roche Applied Science) and 0.1% Triton X-100. The samples were homogenized by 20 strokes with a Dounce homogenizer. After clarification by centrifugation at 17,000× g, samples were loaded onto a black 96-well plate (Packard). Human recombinant MMP-13 (R&D Systems) was included as a positive control. Recombinant MMP-13 and samples were activated by 4-aminophenylmercuric acetate (APMA; Sigma) for 40 min at 37°C. Cleavage of the substrate 5-FAM/QXL™520 FRET peptide was measured in a Wallac Victor 1420 (Perkin Elmer, Waltham, MA).

### Statistical analyses

All statistics were performed in GraphPad Prism Version 4.0 using Student's t-test or Mann-Whitney test. Data is presented as mean ± standard error of the mean (SEM) if not otherwise indicated. To analyze the total number of collagen fibrils counted, the data points (diameter for each fibril) was tabulated in GraphPad using the Frequency distribution analysis tool.

## Supporting Information

Figure S1 **End weights in CT26 and LM3 carcinomas.** **A**) The mean end weight of CT26 tumors from integrin β_3_-deficient and WT mice was 1565±719 mg and 1313±529 mg (n = 22 and 24, respectively; p>0.17). CT26 tumors were harvested 24±5 days after tumor cell injection from integrin β_3_-deficient mice and 23±6 days after tumor cell inoculation from WT mice (p>0.91). Data was analyzed with Student's t-test. Bars are SD. **B**) The mean end weight of LM3 tumors from integrin β_3_-deficient and WT mice was 508±206 mg and 486±165 mg (n = 8 for each genotype; p>0.79). LM3 tumors were harvested 28±6 days after tumor cell injection of integrin β_3_-deficient mice and 28±4 days after tumor cell inoculation of WT mice (p>0.78). Data was analyzed with Mann-Whitney test. Bars are SD.
(TIF)

Figure S2 **Average collagen fibril diameter in CT26 and LM3 carcinomas and in tail dermis.** Mean fibril diameter was calculated for every tumor or tissue from at least three micrographs per sample. **A**) Average fibril diameter in CT26 tumors were 79±13 nm from integrin β_3_-deficient mice and 44±2 nm from WT mice (n = 5 for each genotype; p<0.03). **B**) Average fibril diameter in LM3 tumors were 56±2 nm from integrin β_3_-deficient mice and 47±2 nm from WT mice (n = 3 for each genotype; p<0.05). **C**) Average fibril diameter in tail dermis were 81±7 nm from integrin β_3_-deficient mice and 81±6 nm from WT mice (n = 2 for each genotype; p>0.98). Data was analyzed with Student's t-test. Error bars are SEM.
(TIF)

Figure S3 **Quantitative RT-PCR analyses of mRNAs encoding collagen and lysyl oxidase in LM3 tumors.** Relative mRNA expression of fibrillar pro-collagens and lysyl oxidase (Lox) in LM3 tumors grown in integrin β_3_-deficient (n = 12) compared to WT mice (n = 11). Bars indicate fold changes in tumors grown in integrin β_3_-deficient mice after normalization to tumors from WT mice using the 2^−ΔΔCt^ method. Data was analyzed with Student's t-test. Error bars are SEM.
(TIF)

Figure S4 **Quantitative RT-PCR analyses of mRNAs encoding VEGF-receptor 2, TGF-β1 and TGF-β3, TGF-β-receptor 2 and CTGF in CT26 tumors.** Relative mRNA expression of genes encoding VegfR2,TGF-β1, TGF-β3, TGFβR-II and connective tissue growth factor (CTGF) in CT26 carcinomas from integrin β_3_-deficient (n = 12) compared to WT mice (n = 11). Bars indicate fold changes in tumors grown in integrin β_3_-deficient mice after normalization to tumors from WT mice using the 2^−ΔΔCt^ method. Data was analyzed with Student's t-test. Error bars are SEM.
(TIF)

## References

[1] Jain RK (2005). Normalization of tumor vasculature: an emerging concept in antiangiogenic therapy.. Science.

[2] Lin WW, Karin M (2007). A cytokine-mediated link between innate immunity, inflammation, and cancer.. J Clin Invest.

[3] Lunt SJ, Chaudary N, Hill RP (2009). The tumor microenvironment and metastatic disease.. Clin Exp Metastasis.

[4] Egeblad M, Rasch MG, Weaver VM (2010). Dynamic interplay between the collagen scaffold and tumor evolution.. Curr Opin Cell Biol.

[5] Levental KR, Yu H, Kass L, Lakins JN, Egeblad M (2009). Matrix crosslinking forces tumor progression by enhancing integrin signaling.. Cell.

[6] Barry-Hamilton V, Spangler R, Marshall D, McCauley S, Rodriguez HM (2010). Allosteric inhibition of lysyl oxidase-like-2 impedes the development of a pathologic microenvironment.. Nat Med.

[7] Starling EH (1896). On the absorption of fluids from the connective tissue spaces.. Journal of Physiology.

[8] Reed RK, Liden Å, Rubin K (2010). Edema and fluid dynamics in connective tissue remodelling.. J Mol Cell Cardiol.

[9] Rodt SÅ, Åhlen K, Berg A, Rubin K, Reed RK (1996). A novel physiological function for platelet-derived growth factor-BB in rat dermis.. J Physiol.

[10] Heuchel R, Berg A, Tallquist M, Åhlen K, Reed RK (1999). Platelet-derived growth factor β receptor regulates interstitial fluid homeostasis through phosphatidylinositol-3′ kinase signaling.. Proc Natl Acad Sci U S A.

[11] Nedrebø T, Karlsen TV, Salvesen GS, Reed RK (2004). A novel function of insulin in rat dermis.. J Physiol.

[12] Liden Å, Berg A, Nedrebø T, Reed RK, Rubin K (2006). Platelet-derived growth factor BB-mediated normalization of dermal interstitial fluid pressure after mast cell degranulation depends on β3 but not β1 integrins.. Circ Res.

[13] Svendsen OS, Liden Å, Nedrebø T, Rubin K, Reed RK (2008). Integrin αVβ3 acts downstream of insulin in normalization of interstitial fluid pressure in sepsis and in cell-mediated collagen gel contraction.. Am J Physiol Heart Circ Physiol.

[14] Jain RK (1996). Delivery of molecular medicine to solid tumors.. Science.

[15] Heldin CH, Rubin K, Pietras K, Östman A (2004). High interstitial fluid pressure - an obstacle in cancer therapy.. Nat Rev Cancer.

[16] Tredan O, Galmarini CM, Patel K, Tannock IF (2007). Drug resistance and the solid tumor microenvironment.. J Natl Cancer Inst.

[17] Emerich DF, Snodgrass P, Dean RL, Lafreniere D, Agostino M (2001). Bradykinin modulation of tumor vasculature: I. Activation of B2 receptors increases delivery of chemotherapeutic agents into solid peripheral tumors, enhancing their efficacy.. J Pharmacol Exp Ther.

[18] Pietras K, Rubin K, Sjöblom T, Buchdunger E, Sjöquist M (2002). Inhibition of PDGF receptor signaling in tumor stroma enhances antitumor effect of chemotherapy.. Cancer Res.

[19] Salnikov AV, Iversen VV, Koisti M, Sundberg C, Johansson L (2003). Lowering of tumor interstitial fluid pressure specifically augments efficacy of chemotherapy.. Faseb J.

[20] Salnikov AV, Roswall P, Sundberg C, Gardner H, Heldin NE (2005). Inhibition of TGF-β modulates macrophages and vessel maturation in parallel to a lowering of interstitial fluid pressure in experimental carcinoma.. Lab Invest.

[21] Pietras K, Östman A, Sjöquist M, Buchdunger E, Reed RK (2001). Inhibition of platelet-derived growth factor receptors reduces interstitial hypertension and increases transcapillary transport in tumors.. Cancer Res.

[22] Ozerdem U, Stallcup WB (2003). Early contribution of pericytes to angiogenic sprouting and tube formation.. Angiogenesis.

[23] Liden Å, Karlström A, Lannergård J, Kalamajski S, Guss B (2006). A fibronectin-binding protein from Streptococcus equi binds collagen and modulates cell-mediated collagen gel contraction.. Biochem Biophys Res Commun.

[24] Rubin K, Sjöquist M, Gustafsson AM, Isaksson B, Salvessen G (2000). Lowering of tumoral interstitial fluid pressure by prostaglandin E_1_ is paralleled by an increased uptake of ^51^Cr-EDTA.. Int J Cancer.

[25] Oldberg Å, Kalamajski S, Salnikov AV, Stuhr L, Mörgelin M (2007). Collagen-binding proteoglycan fibromodulin can determine stroma matrix structure and fluid balance in experimental carcinoma.. Proc Natl Acad Sci U S A.

[26] Olive KP, Jacobetz MA, Davidson CJ, Gopinathan A, McIntyre D (2009). Inhibition of Hedgehog signaling enhances delivery of chemotherapy in a mouse model of pancreatic cancer.. Science.

[27] Diop-Frimpong B, Chauhan VP, Krane S, Boucher Y, Jain RK (2011). Losartan inhibits collagen I synthesis and improves the distribution and efficacy of nanotherapeutics in tumors.. Proc Natl Acad Sci U S A.

[28] Varner JA, Cheresh DA (1996). Tumor angiogenesis and the role of vascular cell integrin αVβ3.. Important Adv Oncol.

[29] Hynes RO, Lively JC, McCarty JH, Taverna D, Francis SE (2002). The diverse roles of integrins and their ligands in angiogenesis.. Cold Spring Harb Symp Quant Biol.

[30] Reynolds LE, Wyder L, Lively JC, Taverna D, Robinson SD (2002). Enhanced pathological angiogenesis in mice lacking β3 integrin or β3 and β5 integrins.. Nat Med.

[31] Taverna D, Crowley D, Connolly M, Bronson RT, Hynes RO (2005). A direct test of potential roles for β3 and β5 integrins in growth and metastasis of murine mammary carcinomas.. Cancer Res.

[32] Jin HY, Lee KS, Jin SM, Lee YC (2004). Vascular endothelial growth factor correlates with matrix metalloproteinase-9 in the pleural effusion.. Respir Med.

[33] Tong RT, Boucher Y, Kozin SV, Winkler F, Hicklin DJ (2004). Vascular normalization by vascular endothelial growth factor receptor 2 blockade induces a pressure gradient across the vasculature and improves drug penetration in tumors.. Cancer Res.

[34] Salnikov AV, Heldin NE, Stuhr LB, Wiig H, Gerber H (2006). Inhibition of carcinoma cell-derived VEGF reduces inflammatory characteristics in xenograft carcinoma.. Int J Cancer.

[35] Klosowska-Wardega A, Hasumi Y, Burmakin M, Åhgren A, Stuhr L (2009). Combined anti-angiogenic therapy targeting PDGF and VEGF receptors lowers the interstitial fluid pressure in a murine experimental carcinoma.. PLoS One.

[36] Reynolds LE, Conti FJ, Lucas M, Grose R, Robinson S (2005). Accelerated re-epithelialization in β3-integrin-deficient mice is associated with enhanced TGF-β1 signaling.. Nat Med.

[37] Leask A, Abraham DJ (2006). All in the CCN family: essential matricellular signaling modulators emerge from the bunker.. J Cell Sci.

[38] Taverna D, Moher H, Crowley D, Borsig L, Varki A (2004). Increased primary tumor growth in mice null for β3- or β3/β5-integrins or selectins.. Proc Natl Acad Sci U S A.

[39] Fukumoto Y, Deguchi JO, Libby P, Rabkin-Aikawa E, Sakata Y (2004). Genetically determined resistance to collagenase action augments interstitial collagen accumulation in atherosclerotic plaques.. Circulation.

[40] Brown LF, Papadopoulos-Sergiou A, Berse B, Manseau EJ, Tognazzi K (1994). Osteopontin expression and distribution in human carcinomas.. Am J Pathol.

[41] Urtreger A, Ladeda V, Puricelli L, Rivelli A, Vidal M (1997). Modulation of fibronectin expression and proteolytic activity associated with invasive and metastatic phenotype in two murine mammary tumor cell lines.. Int J Onc.

[42] Hodivala-Dilke KM, McHugh KP, Tsakiris DA, Rayburn H, Crowley D (1999). β3-integrin-deficient mice are a model for Glanzmann thrombasthenia showing placental defects and reduced survival.. J Clin Invest.

[43] Sundberg C, Ljungström M, Lindmark G, Gerdin B, Rubin K (1993). Microvascular pericytes express platelet-derived growth factor-β receptors in human healing wounds and colorectal adenocarcinoma.. Am J Pathol.

[44] Gundersen HJ, Bendtsen TF, Korbo L, Marcussen N, Moller A (1988). Some new, simple and efficient stereological methods and their use in pathological research and diagnosis.. Apmis.

[45] Berg RA (1982). Determination of 3- and 4-hydroxyproline.. Methods Enzymol.

[46] Livak KJ, Schmittgen TD (2001). Analysis of relative gene expression data using real-time quantitative PCR and the 2(−ΔΔC(T)) Method.. Methods.

[47] Ohtani O, Ushiki T, Taguchi T, Kikuta A (1988). Collagen fibrillar networks as skeletal frameworks: a demonstration by cell-maceration/scanning electron microscope method.. Arch Histol Cytol.

