# Supplementary figures and images for: Increased Fibrosis and Interstitial Fluid Pressure in Two Different Types of Syngeneic Murine Carcinoma Grown in Integrin β3-Subunit Deficient Mice
Source: PLoS One. 2012 Mar 30;7(3):e34082. doi: 10.1371/journal.pone.0034082 (PMC3316610; doi:10.1371/journal.pone.0034082)

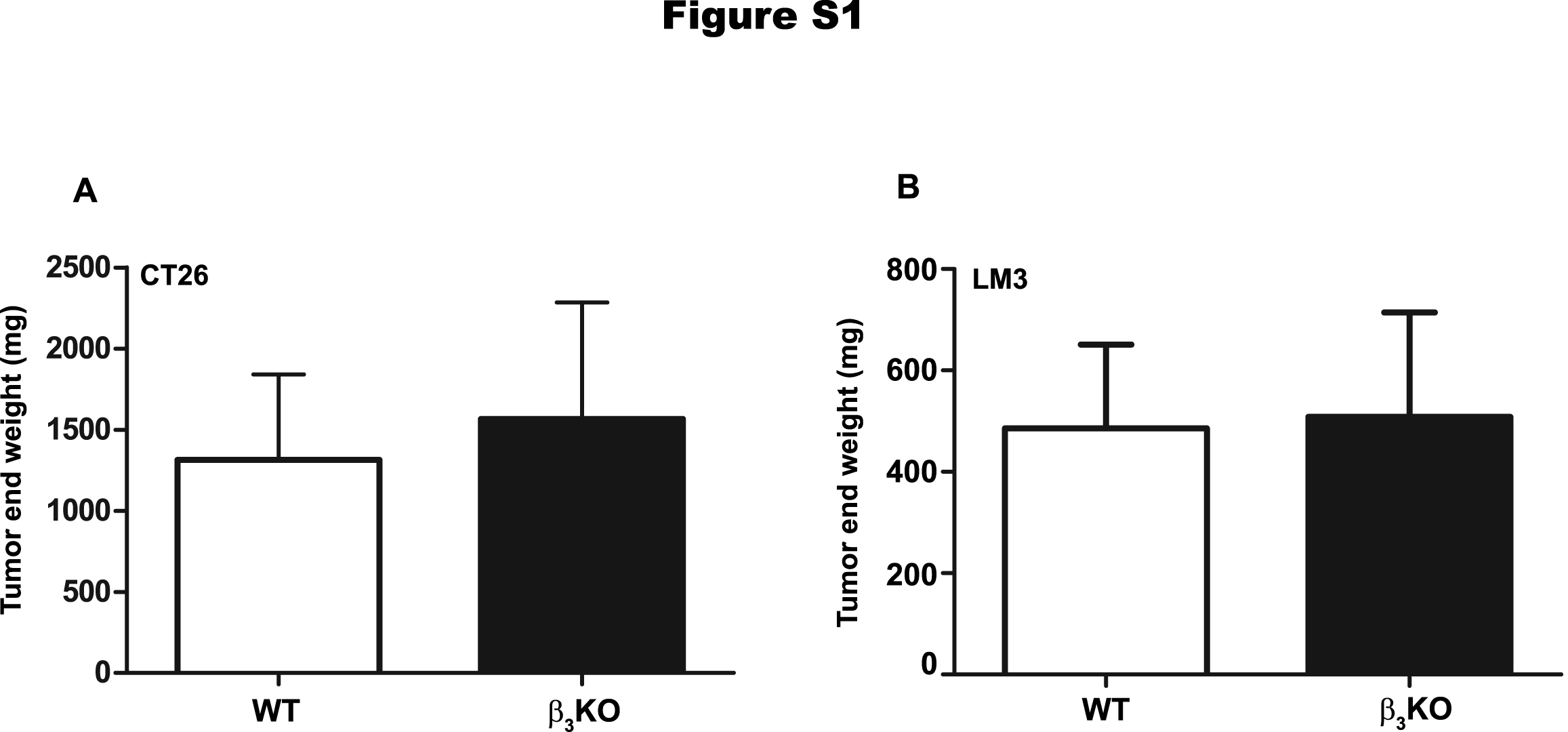

Supplement: Figure S1 — End weights in CT26 and LM3 carcinomas. A) The mean end weight of CT26 tumors from integrin β3-deficient and WT mice was 1565±719 mg and 1313±529 mg (n = 22 and 24, respectively; p>0.17). CT26 tumors were harvested 24±5 days after tumor cell injection from integrin β3-deficient mice and 23±6 days after tumor cell inoculation from WT mice (p>0.91). Data was analyzed with Student's t-test. Bars are SD. B) The mean end weight of LM3 tumors from integrin β3-deficient and WT mice was 508±206 mg and 486±165 mg (n = 8 for each genotype; p>0.79). LM3 tumors were harvested 28±6 days after tumor cell injection of integrin β3-deficient mice and 28±4 days after tumor cell inoculation of WT mice (p>0.78). Data was analyzed with Mann-Whitney test. Bars are SD. (TIF) [file pone.0034082.s001.tif]

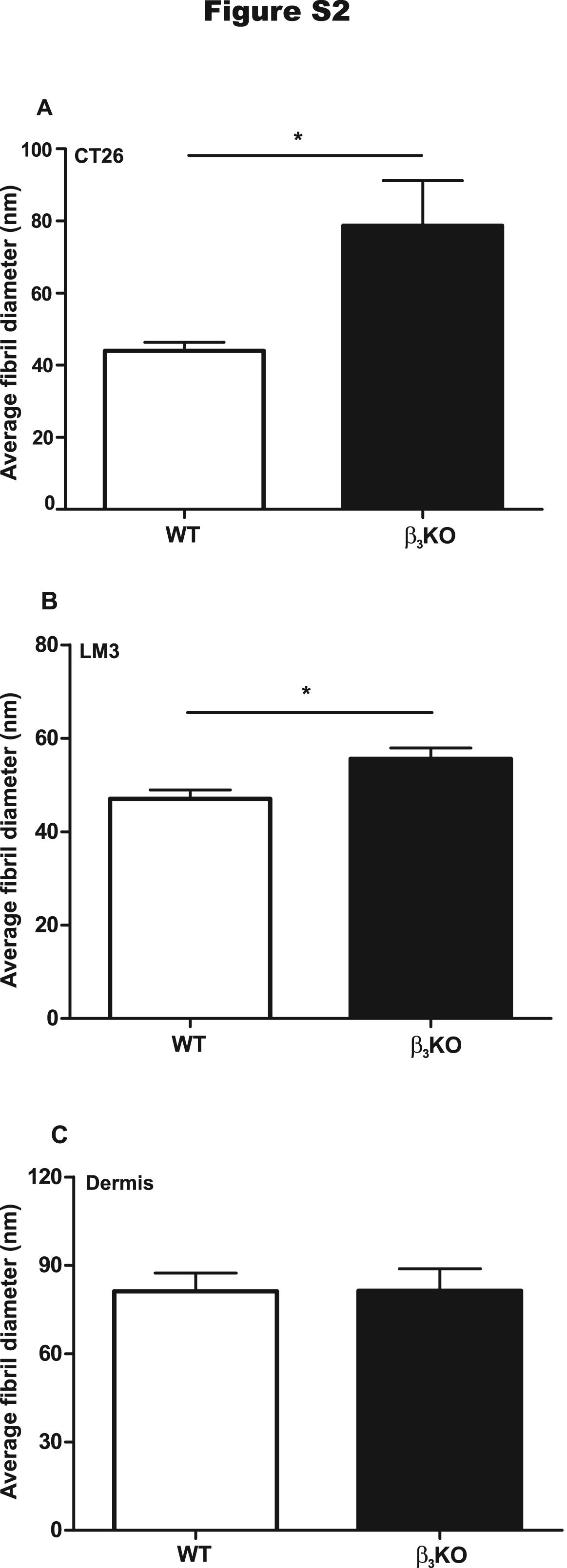

Supplement: Figure S2 — Average collagen fibril diameter in CT26 and LM3 carcinomas and in tail dermis. Mean fibril diameter was calculated for every tumor or tissue from at least three micrographs per sample. A) Average fibril diameter in CT26 tumors were 79±13 nm from integrin β3-deficient mice and 44±2 nm from WT mice (n = 5 for each genotype; p<0.03). B) Average fibril diameter in LM3 tumors were 56±2 nm from integrin β3-deficient mice and 47±2 nm from WT mice (n = 3 for each genotype; p<0.05). C) Average fibril diameter in tail dermis were 81±7 nm from integrin β3-deficient mice and 81±6 nm from WT mice (n = 2 for each genotype; p>0.98). Data was analyzed with Student's t-test. Error bars are SEM. (TIF) [file pone.0034082.s002.tif]

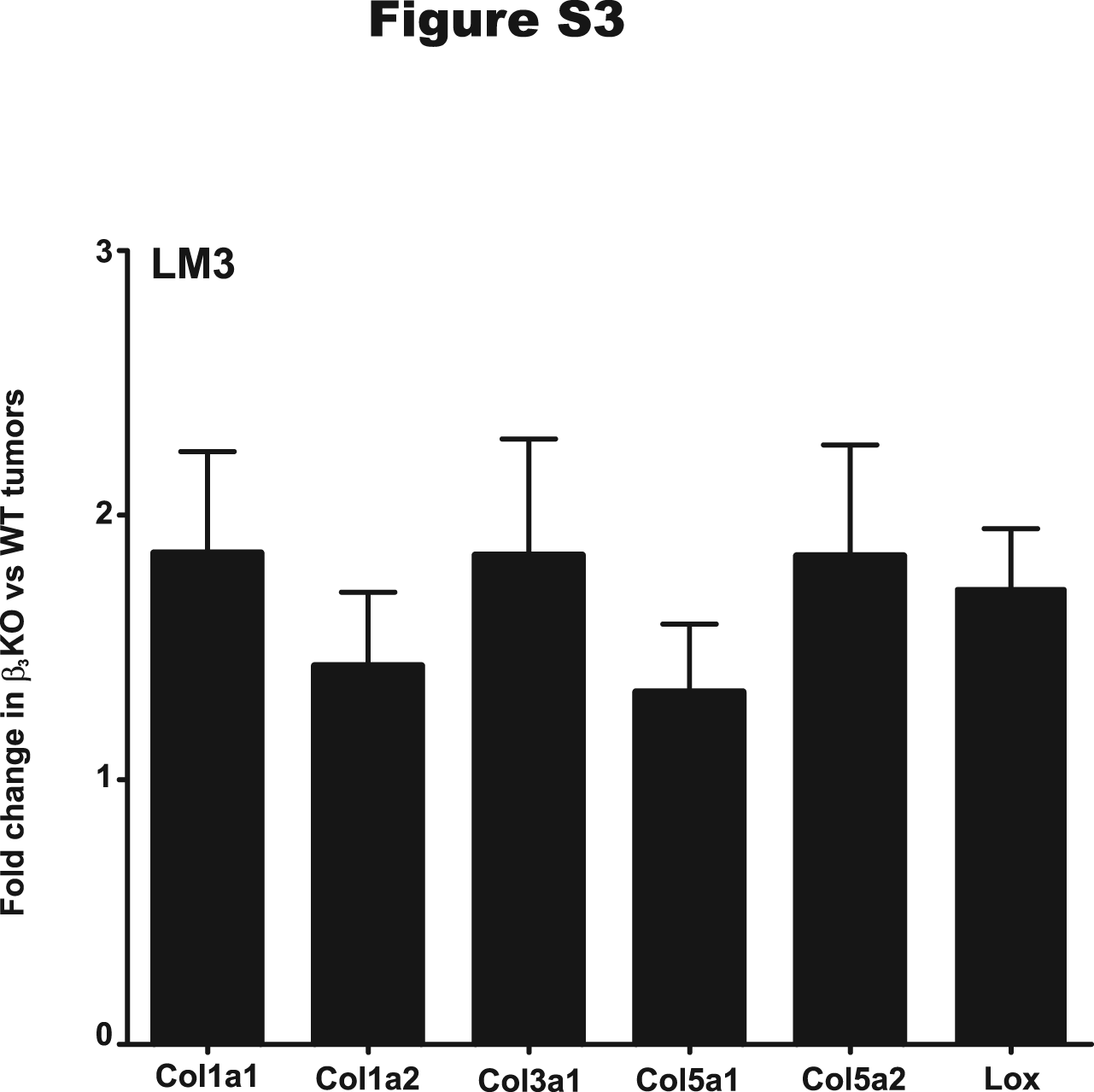

Supplement: Figure S3 — Quantitative RT-PCR analyses of mRNAs encoding collagen and lysyl oxidase in LM3 tumors. Relative mRNA expression of fibrillar pro-collagens and lysyl oxidase (Lox) in LM3 tumors grown in integrin β3-deficient (n = 12) compared to WT mice (n = 11). Bars indicate fold changes in tumors grown in integrin β3-deficient mice after normalization to tumors from WT mice using the 2−ΔΔCt method. Data was analyzed with Student's t-test. Error bars are SEM. (TIF) [file pone.0034082.s003.tif]

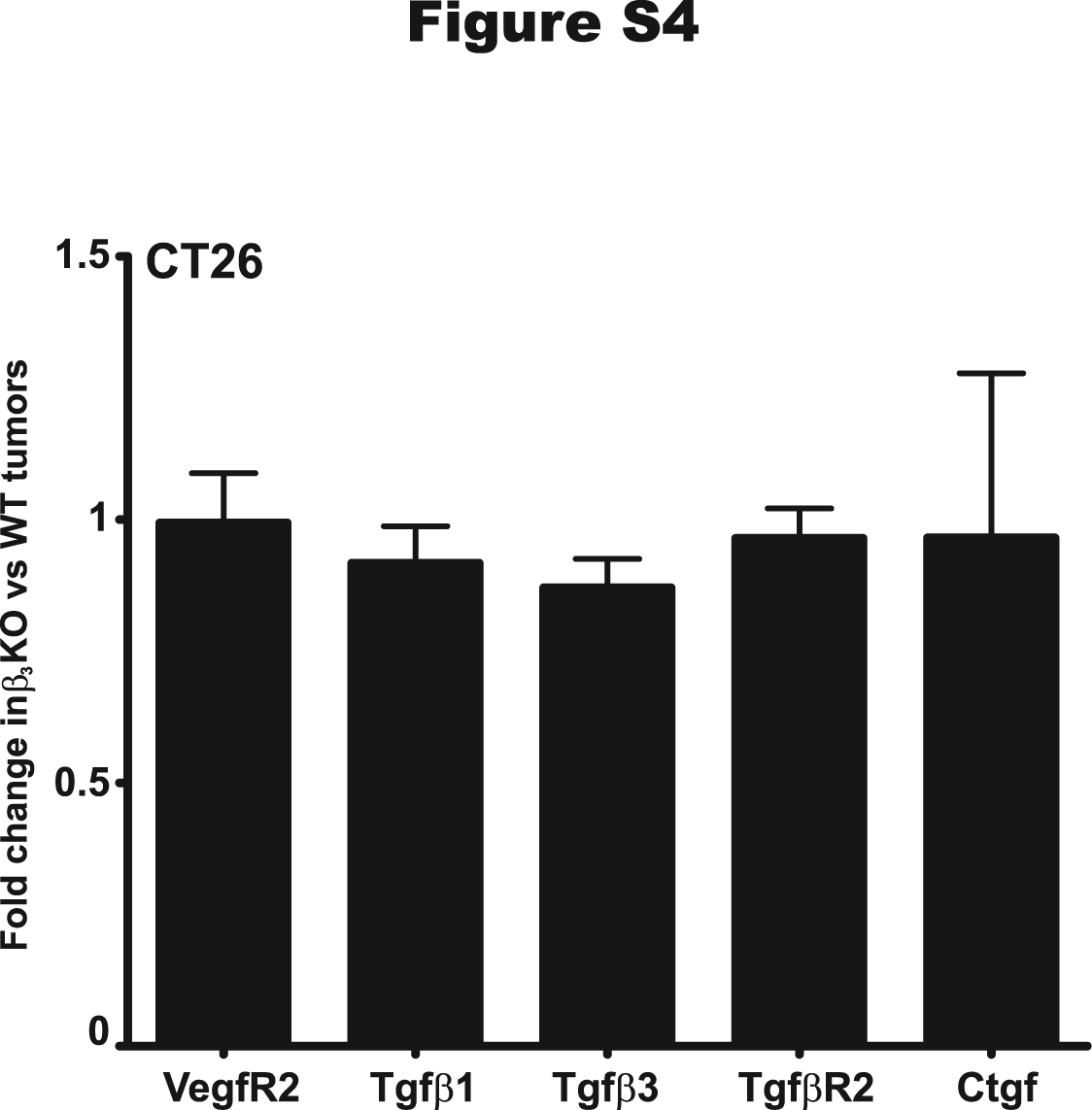

Supplement: Figure S4 — Quantitative RT-PCR analyses of mRNAs encoding VEGF-receptor 2, TGF-β1 and TGF-β3, TGF-β-receptor 2 and CTGF in CT26 tumors. Relative mRNA expression of genes encoding VegfR2,TGF-β1, TGF-β3, TGFβR-II and connective tissue growth factor (CTGF) in CT26 carcinomas from integrin β3-deficient (n = 12) compared to WT mice (n = 11). Bars indicate fold changes in tumors grown in integrin β3-deficient mice after normalization to tumors from WT mice using the 2−ΔΔCt method. Data was analyzed with Student's t-test. Error bars are SEM. (TIF) [file pone.0034082.s004.tif]
